# Mowing of *Carex brevicuspis* (Cyperaceae) improves food quality for herbivorous geese in Dongting Lake: the potential mechanisms

**DOI:** 10.3389/fpls.2025.1566808

**Published:** 2025-05-19

**Authors:** Tao Wu, Le Xia, Baihan Pan, Yeai Zou, Feng Li, Yonghong Xie, Shengze Wang, Zhuoya Li

**Affiliations:** ^1^ College of Life Sciences, Hunan Normal University, Changsha, China; ^2^ Technology Innovation Center for Ecological Conservation and Restoration in Dongting Lake Basin, Ministry of Natural Resources, Hunan Center of Natural Resources Affairs, Changsha, China; ^3^ Institute of Subtropical Agriculture, Chinese Academy of Sciences, Changsha, China; ^4^ National Field Scientific Observation and Research Station of Dongting Lake Wetland Ecosystem in Hunan Province, Changsha, Hunan, China

**Keywords:** hydrological conditions, mowing treatments, *Carex brevicuspis*, bud bank, herbivorous geese, Dongting Lake

## Abstract

The operation of the Three Gorges Dam has altered the hydrological regime of Dongting Lake, leading to earlier water level recession and premature lake bed exposure. The hydrological shift has accelerated the early development of *Carex* spp.-dominated meadows, resulting in phenological mismatches between *Carex brevicuspis* (the primary food source for herbivorous geese) and the arrival of herbivorous geese. This mismatch has reduced suitable food resources for these geese during their wintering period. This study aimed to assess how mowing treatments mitigate this mismatch by improving the quality of *C. brevicuspis*. During the 2023/2024 wintering season, we studied the effects of mowing treatments on *C. brevicuspis* and the underlying mechanisms. Three treatments were applied: complete mowing (CM; stubble height < 0.3 cm), stubble mowing (SM; stubble height 15 cm), and no mowing (CK). The results showed that mowing treatments significantly increased leaf moisture and nitrogen content, reduced crude fiber content, and thereby increased the quality and food utilization rates by herbivorous geese. Additionally, mowing increased the quality of *C. brevicuspis* by stimulating compensatory growth responses, which relies on the resources of the belowground bud banks mechanisms. However, mowing treatments also significantly decreased plant height, density, aboveground biomass, and the biomass of belowground bud banks, which potentially impairs the long-term recovery and sustainability of plant. Therefore, while targeted mowing can effectively improve the food quality for herbivorous geese during wintering, careful consideration of the management of mowing frequency and intensity are essential to maintain balance between the wetland vegetation recovery and the sustainable food supply for herbivorous geese.

## Graphical Abstract

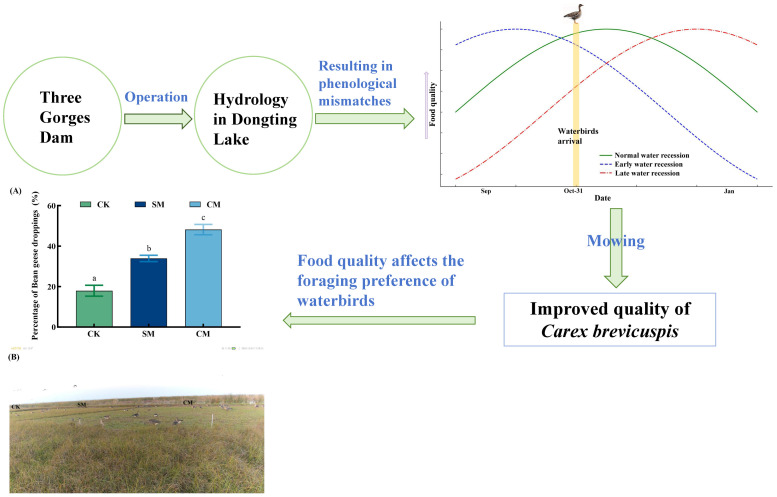

## Highlights

- Mowing significantly improves the nutritional quality of *C. brevicuspis*, increasing food digestibility and palatability, thereby increasing its utilization by herbivorous geese.
- Mowing increases leaf nitrogen content and reduce crude fiber content in *C. brevicuspis* through compensatory growth responses and mediation by belowground bud bank mechanisms.
- Although mowing effectively improves food quality, it simultaneously reduces aboveground biomass and bud biomass, highlighting the necessity of carefully managing mowing intensity and frequency to balance plant regeneration and sustainable habitat management.

## 1 Introduction

The nutritional composition of food represents a key factor in governing the dietary and habitat selection strategies of herbivores (Washburn and Seamans, 2012). Plant nutrients, particularly protein levels, strongly dictate the food preferences of herbivores (Wang et al., 2024). Herbivorous geese preferentially forage in meadows abundant in high-quality food (Van Der Graaf et al., 2007). For herbivorous geese, food quality critically determines their survival, reproductive success, and migratory performance, by influencing their energy reserves and overall physical condition. Key attributes of foods, such as the nutritional composition, energy density, and palatability, determine whether migratory birds can efficiently achieve the nutrients they need for migration. As plants grow and mature, their height, biomass, and nutritional components (e.g., protein and fiber content) undergo dynamic shifts. Declining food quality and insufficient nutrient availability can negatively affect the survival and behavior of herbivorous geese (Wang et al., 2013; Zhang et al., 2020).

Phenological mismatch refers to the temporal misalignment between the peak nutrient demand of animals and the availability of optimal food resources (Durant et al., 2007). Migratory herbivorous geese are particularly susceptible to this phenomenon (Beard et al., 2019). A striking case occurred in East Dongting Lake, a Ramsar site along the East Asian-Australasian Flyway, large numbers of herbivorous geese for stopover and overwintering every year (Zou et al., 2017) and supports herbivorous geese with abundant food resources through its rich wetland vegetation (Feng et al., 2014; Zhang et al., 2023). However, human activities and altered hydrological regimes have significantly changed the ecological patterns of the wetland. Specifically, the operation of the Three Gorges Dam has advanced the autumn water recession time (Wu et al., 2017; Cheng et al., 2018), prematurely exposing sedge meadows and promoting early senescence of *C. brevicuspis* as the dominant species (Feng et al., 2013; Lai et al., 2013). This mismatch results in migratory birds relying on suboptimal food, further exacerbating their foraging and survival challenges (Zhang et al., 2021).

Providing high-quality habitats for herbivorous geese is a fundamental aspect of wetland conservation (Murray and Fuller, 2015; O’neal et al., 2008). Addressing the negative impacts of hydrological changes on wetland ecosystems has become a critical issue in the current context. As a targeted management measure, mowing could effectively regulate the growth of wetland vegetation and alleviate the ecological pressures brought about by hydrological changes (Zhao et al., 2008). Mowing removes specific vegetation, regulating plant height and density, and altering species composition, thereby creating favorable foraging conditions for herbivorous geese. These modifications collectively enhance habitat suitability for herbivorous geese by optimizing foraging microhabitats, reducing energy expenditure during feeding activities (Zuo et al., 2018). Moreover, appropriate mowing improves plant quality (Mikhailova et al., 2000; Gardarin et al., 2014), particularly by increasing nitrogen content and reducing crude fiber content, which increases food digestibility and palatability (Veloso and Bozinovic, 1993). These changes increase habitat carrying capacity (Kołos and Banaszuk, 2013; Molina et al., 2021; Van De Koppel et al., 1996), ensuring sufficient food availability for waterbird populations while maintaining critical wetland functions.

The effects of mowing on *C. brevicuspis* extend beyond aboveground part, exerting substantial impacts on belowground bud banks (Chen and Deng, 2016). Clonal plants such as *C. brevicuspis* mainly propagate through belowground bud banks (Benson et al., 2004; Sosnová et al., 2010; Deng et al., 2015), with bud germination and tillering are critical for plants to cope with disturbances and stresses. Morphological classification based on rhizome architecture identifies two distinct bud types: long rhizome buds (LRB) and short rhizome buds (SRB) (Chen et al., 2011; Chen and Deng, 2016). Long rhizome buds extend horizontally, facilitating the guerrilla growth form to escape resource-poor or competitive environments. In contrast, short rhizome buds grow vertically, supporting resource-rich environments through a phalanx growth strategy (Chen and Deng, 2016; Ott and Hartnett, 2015). When facing mowing stress, the plasticity of these clonal growth forms allows plants to adapt to various environmental pressures, such as transitioning from phalanx to guerrilla growth, aiding in population recovery and expansion (N’Guessan et al., 2011; Ott and Hartnett, 2015). Consequently, mowing response of *C. brevicuspis* is intrinsically linked to belowground bud banks (Huhta et al., 2000; Ott and Hartnett, 2015). This study aims to elucidate how mowing optimizes the synergy between aboveground and belowground plant components to improve the food quality of *C. brevicuspis*, providing more nutritious and digestible food resources for herbivorous geese.

While hydrological impacts on wetland vegetation-geese interactions are well documented (Teng et al., 2023; Liu et al., 2024a), mechanistic understanding of mowing-mediated vegetation quality regulation remains limited. This study addresses three critical questions through systematic analysis of *C. brevicuspis*: (1) How does mowing affect the food quality of *C. brevicuspis*, and what are the mechanisms underlie these changes? (2) What are the effects of mowing treatments on the belowground bud banks of *C. brevicuspis*, and how do these changes impact the plant’s long-term recovery and resilience to mowing pressures? (3) What ecological management implications can be drawn from the mowing experiments?

## 2 Materials and methods

### 2.1 Study area

**Figure 1:**
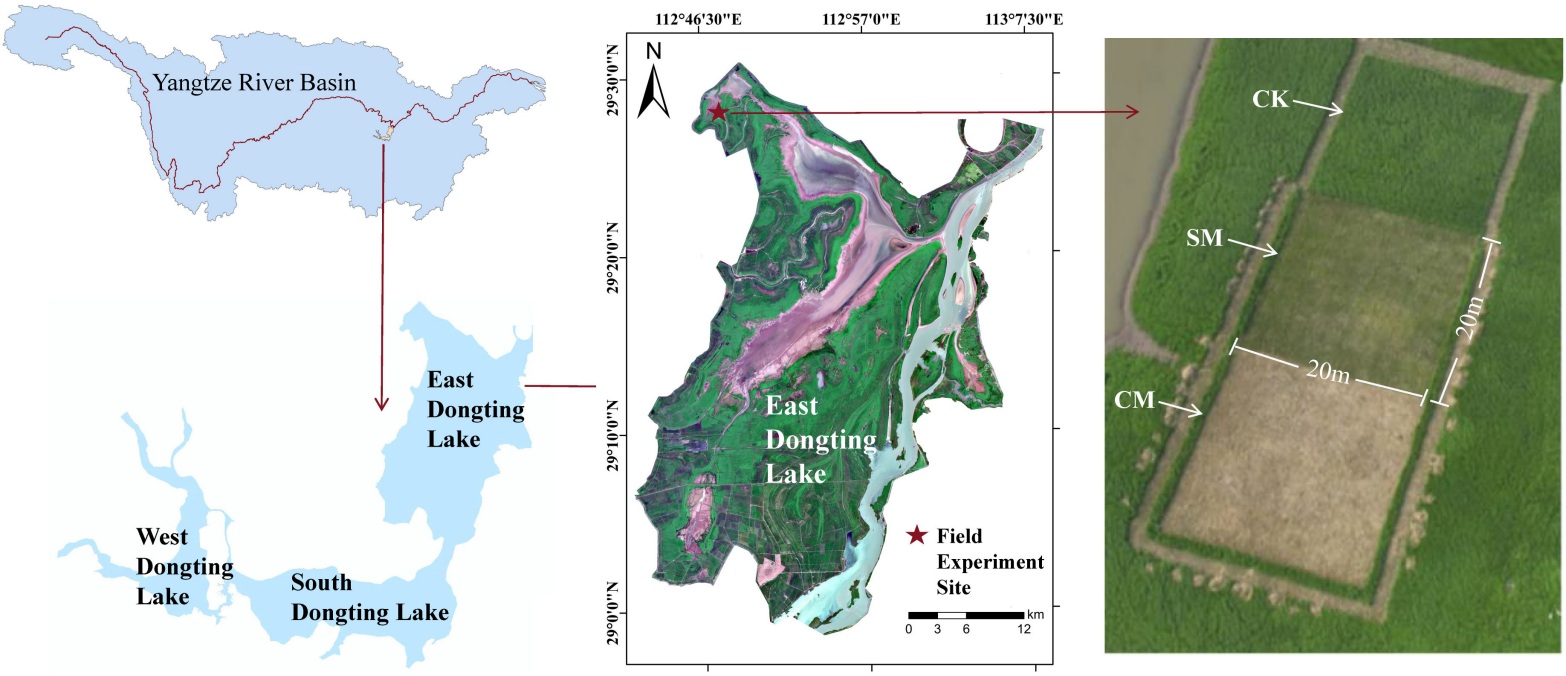
The site location and experimental design of the mowing control experiment, accompanied by an aerial photograph of one of the experimental transects on the right.

Dongting Lake (28°30′-30°20′N, 111°40′-113°10′E), a quintessential example of a river-linked lake system in the Yangtze River’s mid-reaches, comprises three interconnected sub-basins: East, South, and West Dongting Lake (**Figure 1**). Spanning 2,625 km² after hydrological adjustments and anthropogenic modifications, it plays a vital role as a regulating lake in the Yangtze River Basin (Xie et al., 2015). The lake exhibits pronounced seasonal hydrological dynamics, with water levels fluctuating from summer peaks near 36 m to winter troughs under 20 m (Zhang et al., 2020). The operation of the Three Gorges Dam has altered the hydrological regime of Dongting Lake, leading to earlier water level recession and premature lake bed exposure (Gao et al., 2020).

East Dongting Lake, the major part of Dongting Lake, has been recognized as the key wintering region in Yangtze River floodplain for hundreds of thousands of migratory waterbirds of the East Asian-Australasian Flyway (Cao et al., 2008). This region hosts over 90% of the geese occurring in Dongting Lake (Cao et al., 2008; Wang et al., 2012), with dominated species including the Eastern Tundra Bean Goose (maximum number, 42,164), Lesser White-fronted Goose (maximum number, 24,261), and Greater White-fronted Goose (maximum number, 12,275) (Zou et al., 2017). This study was conducted at Xiaoxi Lake, located within the East Dongting Lake Wetland Nature Reserve.

**Figure 2:**
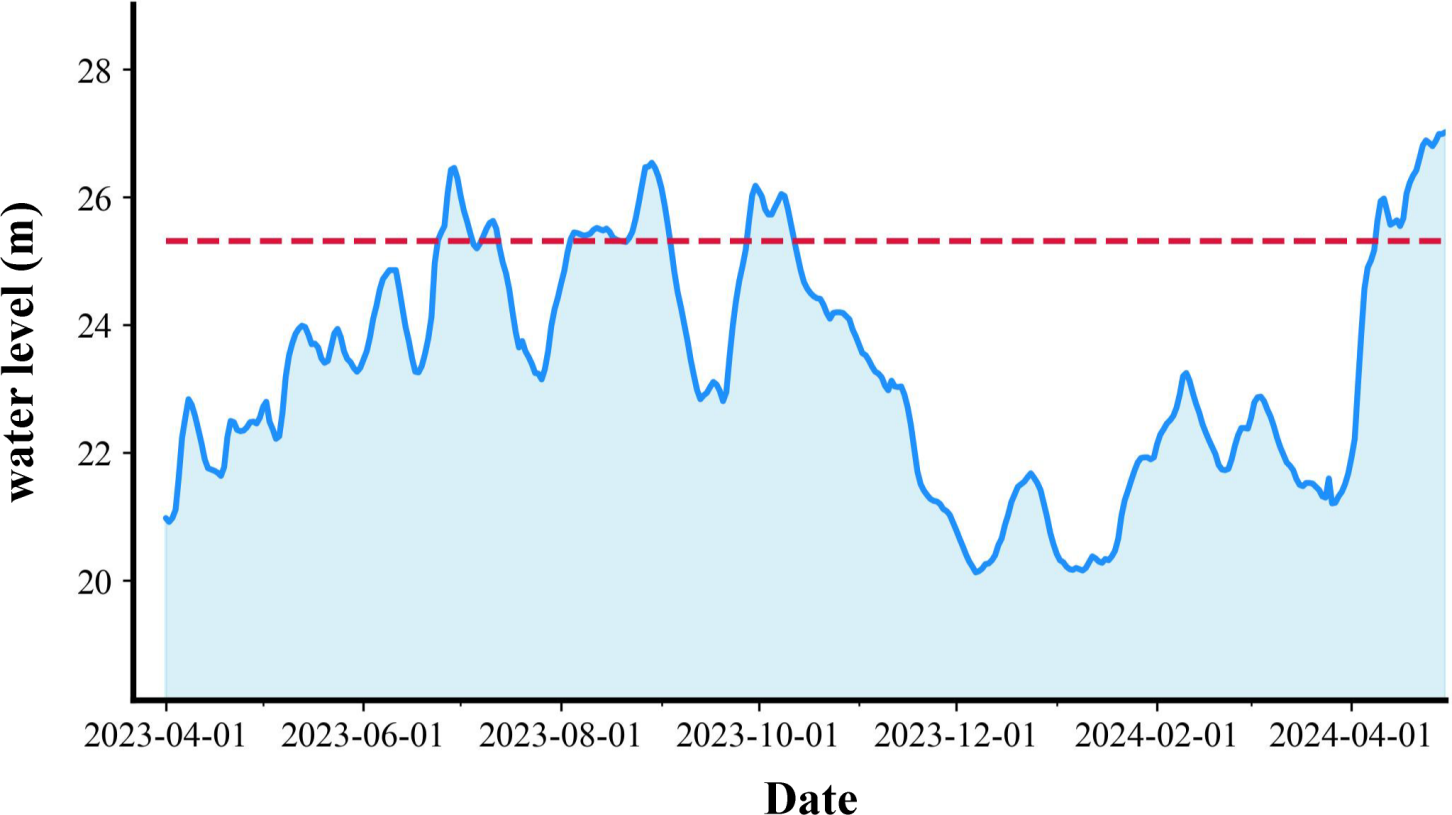
Variations in water level from early April 2023 to early April 2024. The dash line in the upper panels denotes water level of 25.31 m, at which the *Carex* spp. meadows are entirely exposed (Zhang et al., 2021).

This study acquired daily water level data at 08:00 for Dongting Lake were obtained from the Hydrology Inquiry System of Hunan Province, systematically obtaining a complete hydrological data (April 2023 to April 2024) daily water level time-series dataset (**Figure 2**). According to previous data (Zhang et al., 2021), the water level at which meadows are completely exposed is 25.31 m. Based on historical hydrological analysis (Zhang et al., 2021), the 2023/2024 overwintering period can be defined as early water recession.

### 2.2 Experimental design

The experiment was conducted at the field comprehensive observation site of the Dongting Lake Wetland Ecosystem Research Station, Chinese Academy of Sciences (29°30′ N, 112°48′ E, **Figure 1**). In October 2023, when the sedge meadows were fully exposed in Xiaoxi Lake, typical *C. brevicuspis* communities were selected in areas with no human disturbances, uniform distribution, and consistent elevation. Three parallel transects dominated by *C. brevicuspis* were established, near and parallel to the water’s edge. Each transect included three mowing treatments: complete mowing (CM; stubble height < 0.3 cm), stubble mowing (SM; stubble height 15 cm), and no mowing (CK) (**Figure 1**). Each transect consisted of three quadrats of equal area (each 20 m × 20 m).

### 2.3 Study species

The *Carex* vegetation, composed mainly of *C. brevicuspis* and other species, is one of the dominant vegetation types in Dongting Lake (Xie et al., 2015), widely distributed on meadows with an elevation of 24–26 meters. *C. brevicuspis*, a perennial herbaceous plant of the genus *Carex* in the Cyperaceae family, serves as the crucial food for overwintering herbivorous geese in Dongting Lake due to its phenological synchronization with these geese migration (Wang et al., 2019). The life history strategy of *C. brevicuspis* is closely related to hydrological changes, particularly the timing of the autumn water recession in Dongting Lake. Seasonal flooding from June to October induces vegetative dormancy, with complete submergence of belowground rhizomes. When water recedes in Dongting Lake, *C. brevicuspis* germinates rapidly and completes its first growth cycle by January. As temperatures decrease, *C. brevicuspis* gradually turns yellow and enters dormancy. New clonal ramets emerge in spring, marking the beginning of the second growing season. The plants grow rapidly and bear fruit before the next flood, which typically occurs in April or May of the following year, thus completing their life cycle (Deng et al., 2013b; Chen et al., 2014).

### 2.4 Sample collection and processing

Sample collection was conducted from late October 2023 to March 2024, with sampling occurring twice per month (8th and 23rd of each month), encompassing 11 sampling events (T0-T10). The first sampling on October 23 was designated as T0, and the subsequent samplings on November 8, December 23, and so on, were recorded sequentially up to T10 on March 23. T0 represents the pre-mowing sampling date for *C. brevicuspis*, and T1 represents the post-mowing sampling date, with the first growth cycle ending at T6 and the second growth cycle beginning at T7. During each sampling event, six 1 m × 1 m quadrats were randomly established for each treatment, resulting in 18 quadrats per sampling. Within the quadrats, the density and height of *C. brevicuspis* were recorded. All living aboveground parts of the plants (>50% green, potentially photosynthetically active) were harvested, brought back to the laboratory, dried, and weighed to determine biomass and nutrient content (nitrogen and crude fiber). Simultaneously, the belowground parts of the plants were excavated (ensuring the connections between belowground structures were retained), brought back to the laboratory, carefully cleaned, and categorized into bud types (LRB and SRB). The total rhizome buds (TRB) density was calculated as the sum of the LRB and SRB per square meter. The belowground parts were then dried, and their biomass was recorded. Herbivorous geese, the target animals, often rest and defecate near their feeding sites, and the number of droppings can reflect the number of herbivorous geese feeding in the quadrats and their food preferences to some extent. To ensure data accuracy, all droppings within the quadrats were removed after each count.

### 2.5 Statistical analysis

The differences in *C. brevicuspis* population characteristics and nutritional levels under different mowing treatments were tested using repeated measures ANOVA, with mowing treatment as the main factor, sampling period as the repeated measure, and transects set as a covariate to eliminate the influence of experimental transects in result comparisons. For data that did not conform to a normal distribution, a Mixed Linear Model was applied to examine the effects of mowing on plant traits in *C. brevicuspis* populations. As no rhizome buds were produced after late February, the belowground bud banks were not analyzed beyond this period. A one-way ANOVA was conducted to test the differences in *C. brevicuspis* utilized by herbivorous geese under different mowing treatments. Multiple comparisons of the means of plant traits under three mowing treatments at each sampling period were performed using Tukey’s honest significant difference (HSD) test at a 0.05 significance level. Some of the data were square root- or log10-transformed to reduce the variance heterogeneity, and the homogeneity was tested using Levene’s test. In all the statistical results, statistical significance was set at *p* < 0.05. All analyses were conducted in R ver. 4.1.2.

## 3 Results

### 3.1 Effects of mowing on food quality for herbivorous geese

#### 3.1.1 Moisture content, nitrogen content, and crude fiber content

**Figure 3:**
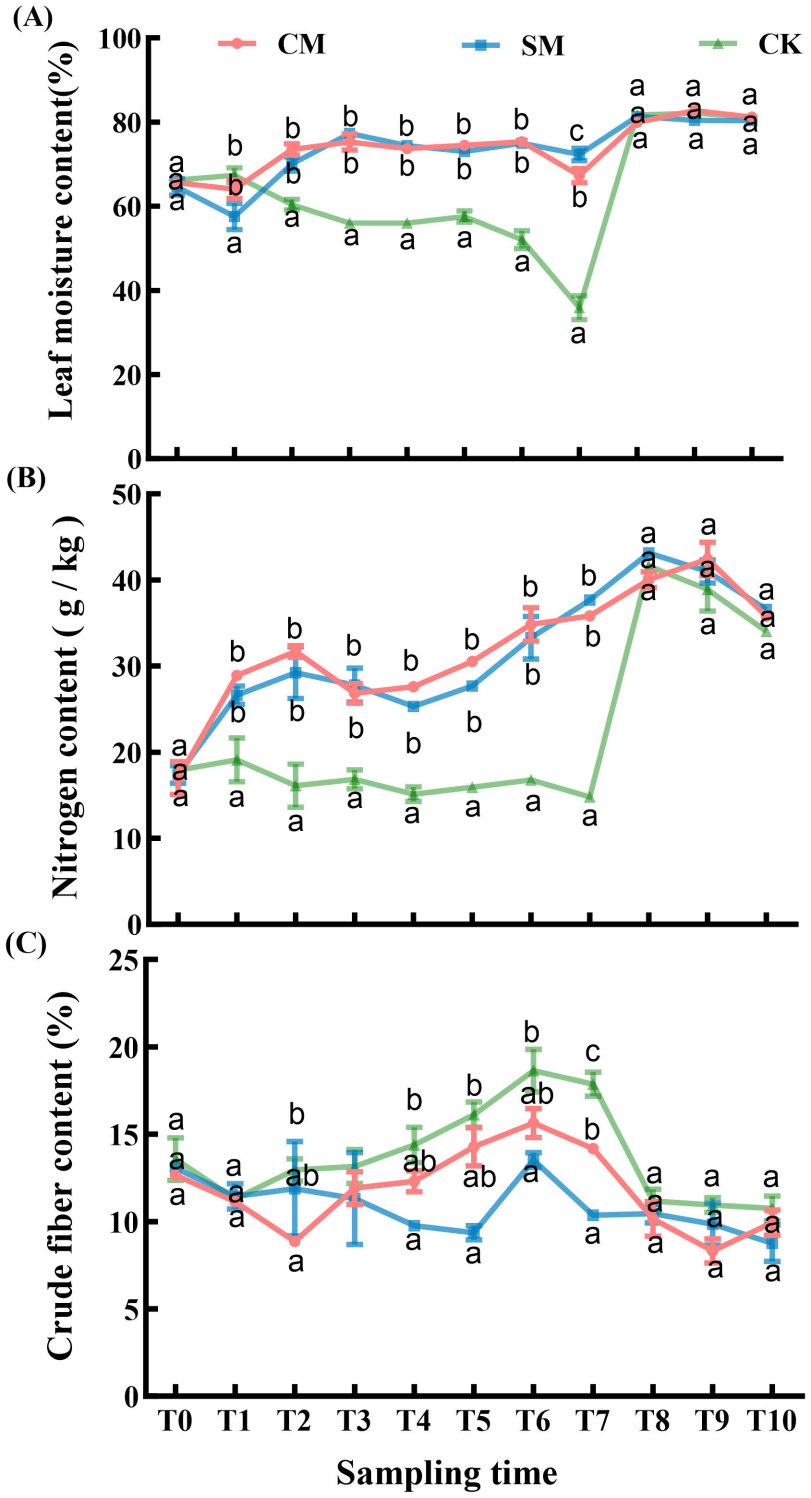
**(A)** Leaf moisture content, **(B)** nitrogen content, and **(C)** crude fiber content of *Carex. brevicuspis* under different mowing treatments, October 2023-March 2024. The data are expressed as the mean ± standard error (SE). Different letters indicate statistically significant differences (Tukey HSD test of pairwise comparisons).

Mowing treatments and sampling time exerted significant effects on leaf moisture content of *C. brevicuspis* (**Table 1**). Specifically, mowing treatments significantly increased the leaf moisture content (*p* < 0.001, **Figure 3A**). Both CM and SM treatments maintained elevated moisture levels throughout the overwintering period (**Figure 3A**), whereas CK treatment exhibited pronounced decline during T5-T7 (*p* < 0.001, **Figure 3A**), reaching a nadir at T7, followed by a rapid recovery until the three treatment groups converged.

**Table 1:** The linear mixed model on plant traits in *C. brevicuspis* populations for different mowing treatments.

Variable	Mowing treatments (M)	Sampling time (S)	M × S
Sward height	<0.001^***^	<0.001^***^	<0.001^***^
density	<0.001^***^	<0.001^***^	<0.001^***^
Aboveground biomass	<0.001^***^	<0.001^***^	<0.001^***^
Moisture content	<0.001^***^	<0.001^***^	<0.001^***^
Biomass per LRB	0.004^**^	<0.001^***^	0.005^**^

Asterisks denote significant levels (*** *p* < 0.001; ** *p* < 0.01).

Values in the table are the corresponding p-values.

Nitrogen content was significantly affected by both mowing treatments and sampling time (**Table 2**). Mowing treatments significantly enhanced the nitrogen content in the leaves of *C. brevicuspis* (*p* < 0.001, **Figure 3B**), with no divergence between CM and SM treatments. CK treatment consistently showed lower nitrogen, though treatment differences attenuated by T8-T10.

**Table 2:** Summary of repeated ANOVAs on plant traits in *C. brevicuspis* populations for different mowing treatments (*F* and *P*-values).

Variable	Mowing treatments (M)	Sampling time (S)	M × S
Nitrogen content^a^	250.957^***^	92.484^***^	10.217^**^
Crude fiber content^a^	14.682^*^	12.323^**^	2.734 ^ns^
Root biomass^b^	0.888^ns^	2.862^**^	0.788^ns^
TRB density	2.185^ns^	3.844^**^	1.310^ns^
SRB density	1.948^ns^	3.378^**^	1.062^ns^
LRB density	1.434^ns^	3.587^**^	1.411^ns^
Biomass per SRB	16.197^***^	3.356^**^	1.455^ns^
SRB proportion	0.402^ns^	1.360 ^ns^	1.751^ns^
d.f.	2	5	10

Asterisks denote significant levels (*** *p* < 0.001; ** *p* < 0.01; * *p* < 0.05; ns *p* > 0.05).

^a^d.f. for sampling time (S) is 10 and M × S is 20; ^b^d.f. for sampling time (S) is 8 and M × S is 16.

Crude fiber content was significantly affected by both mowing treatments and sampling time (**Table 2**). SM treatment significantly reduced crude fiber content in the leaves of *C. brevicuspis* (*p* < 0.05, **Figure 3C**). Although the differences between CM and CK did not reach the traditional significance level (*p*=0.057), the observed trends suggest that the CM treatment may exert some influence on the crude fiber content of *C. brevicuspis*. However, no significant difference was observed between CM and SM treatments (*p* > 0.05).

### 3.2 Effects of mowing on population characteristics of *C. brevicuspis*

#### 3.2.1 Sward height and density

**Figure 4:**
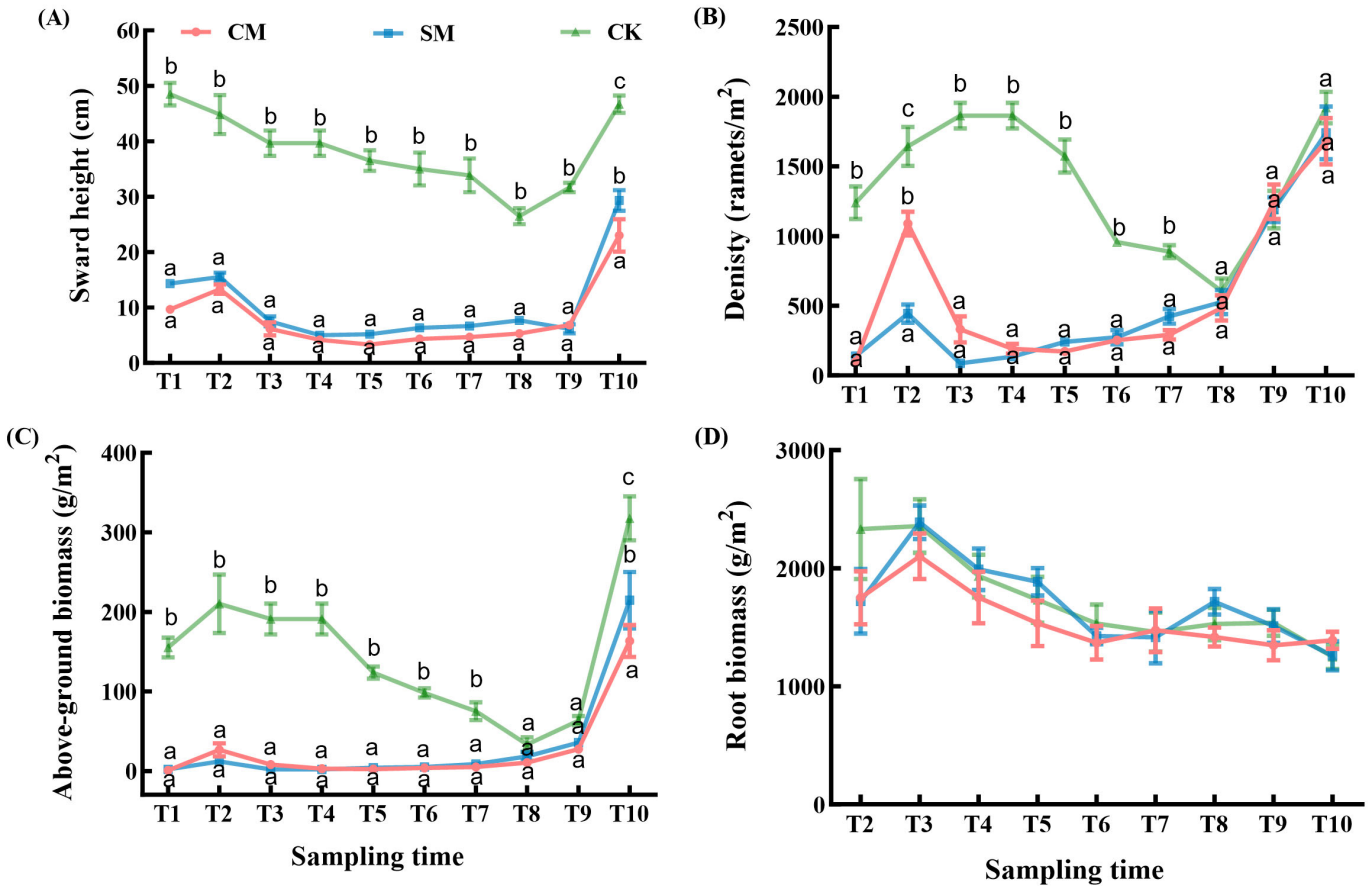
**(A)** Sward height, **(B)** density, **(C)** above-ground biomass, and **(D)** root biomass of *C*. *brevicuspis* under different mowing treatments, November 2023-March 2024. The data are expressed as the mean ± standard error (SE). Different letters indicate statistically significant differences (Tukey HSD test of pairwise comparisons).

Mowing treatments and sampling time imposed strong suppressive effects on the height and density of *C. brevicuspis* (**Table 1**). However, there were no significant differences in height and density between CM and SM treatments (*p* > 0.05, **Figures 4A, B**). Notably, in the second growing season, the height of *C. brevicuspis* under both mowing treatments was significantly lower than CK treatment (*p* < 0.001, **Figure 4A**), while the density showed no significant difference (*p* > 0.05, **Figure 4B**).

#### 3.2.2 Aboveground biomass and root biomass

Both mowing treatments and sampling time had highly significant effects on the aboveground biomass of *C. brevicuspis* (**Table 1**). Mowing treatments significantly reduced the aboveground biomass (*p* < 0.001, **Figure 4C**), with both CM and SM treatments exhibiting similar trends. The aboveground biomass remained at low levels from T1 to T8 and increased rapidly at T9 and T10.

The root biomass of *C. brevicuspis* was not significantly affected by mowing treatments (**Table 2**), but it was significantly affected by sampling time (*p* < 0.01, **Table 2**). The trends in root biomass under different treatments were relatively similar. Although no significant differences were observed at the α = 0.05 level, the root biomass in CM treatment was slightly lower than in the other treatment groups (**Figure 4D**).

### 3.3 Effects of mowing on the belowground Bud banks of *C. brevicuspis*

#### 3.3.1 Changes in the density of rhizome buds of *C. brevicuspis*

**Figure 5:**
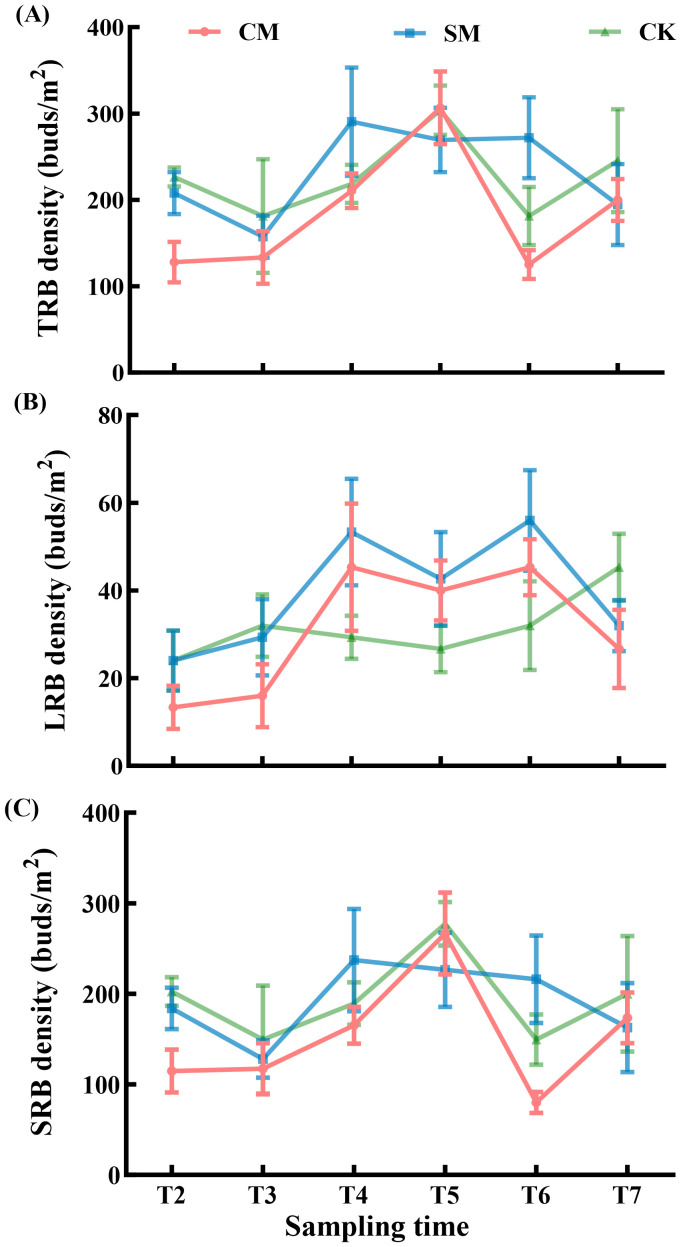
**(A)** Total rhizome buds (TRB), **(B)** long rhizome buds (LRB), and **(C)** Short rhizome buds (SRB) density of *C. brevicuspis* populations under different mowing treatments, November 2023-February 2024. Different scales are used on the y-axis. The data are expressed as the mean ± standard error (SE).

**Figure 6:**
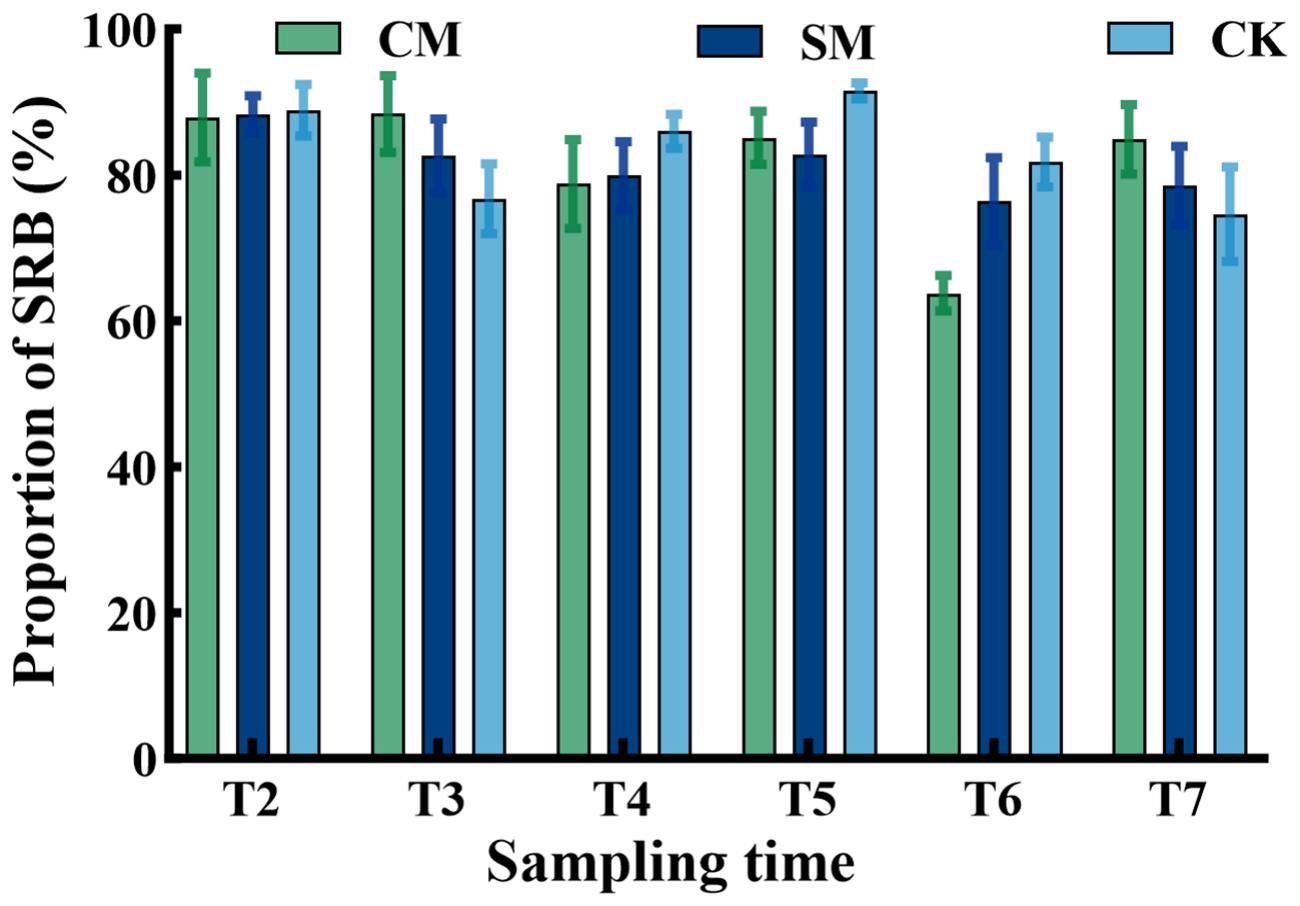
The proportion of short rhizome buds (SRB) of *C. brevicuspis* under different mowing treatments, November 2023-February 2024. The data are expressed as the mean ± standard error (SE).

The sampling time had a significant effect on the TRB density, as well as SRB density and LRB density (**Table 2**), however, the density of rhizome buds was not significantly affected by the mowing treatments (**Figures 5A–C**). Most of the rhizome buds of *C. brevicuspis* were composed of SRB (**Figure 6**), and the ratio of SRB to LRB was not affected by either mowing treatments or sampling time (*p* > 0.05, **Table 1**). The changes in the SRB and TRB densities displayed similar trends for all treatments (**Figures 5A, B**).

#### 3.3.2 Changes in the biomass of rhizome buds

**Figure 7:**
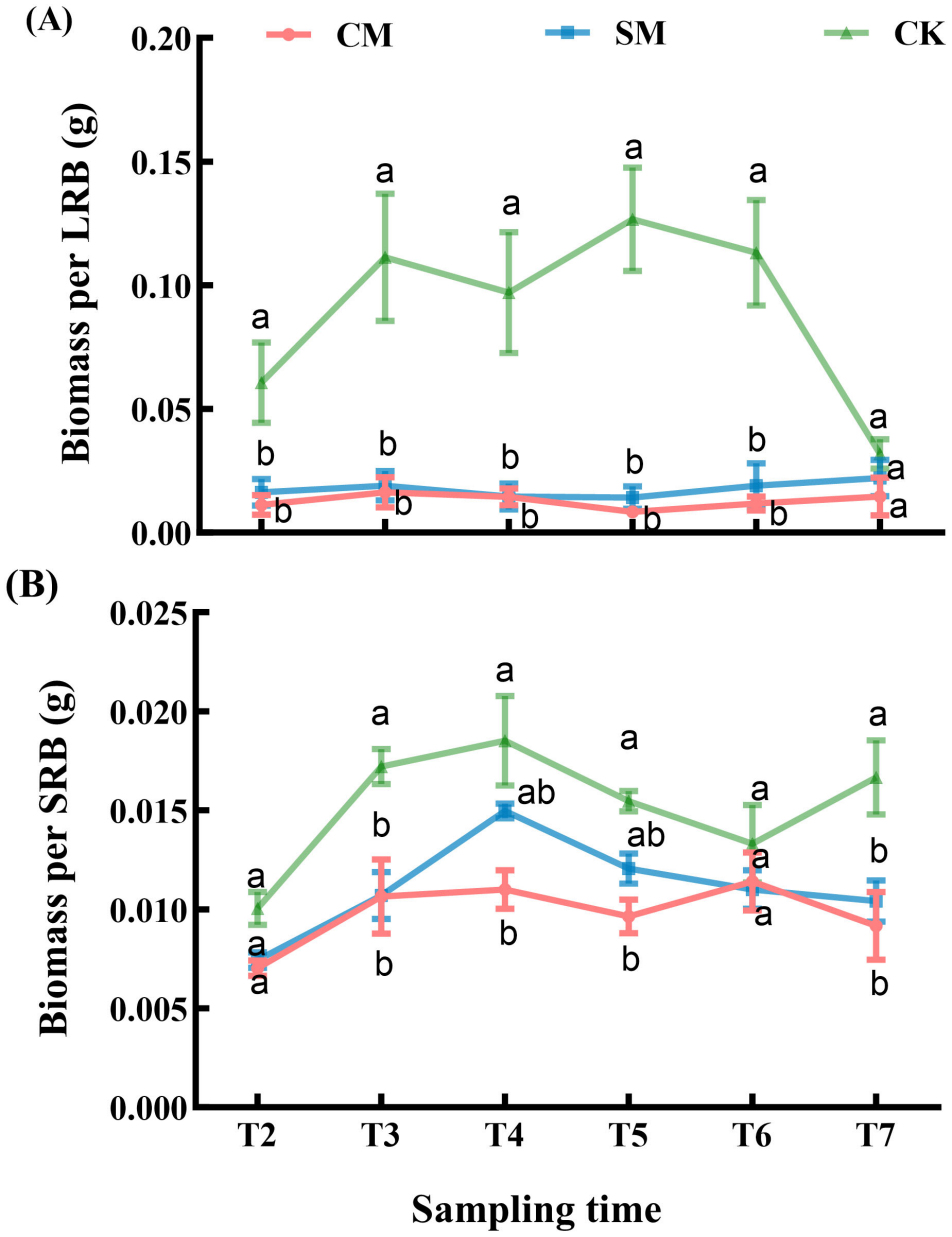
**(A)** Biomass per long rhizome buds (LRB) and **(B)** short rhizome buds (SRB) of C. *brevicuspis* populations under different mowing treatments, November 2023-February 2024. The data are expressed as the mean ± standard error (SE). Different letters indicate statistically significant differences (Tukey HSD test of pairwise comparisons).

The sampling time had a significant effect on the biomass of rhizome buds, mowing treatments significantly reduced the biomass of LRB (**Table 1**), while there was no significant difference between CM and SM treatments (*p* > 0.05, **Figure 7A**). The biomass of SRB was significantly affected by both mowing treatments and sampling time (**Table 2**). Mowing significantly reduced the biomass of SRB (*p* < 0.001, **Figure 7B**). The biomass in CM treatment was significantly lower than in CK treatment (*p* < 0.001), and SM treatment was also lower than CK treatment (*p* < 0.001), no significant difference was observed between CM and SM treatments (*p* > 0.05, **Figure 7B**).

### 3.4 Effects of mowing on utilization of *C. brevicuspis by* Bean geese

**Figure 8:**
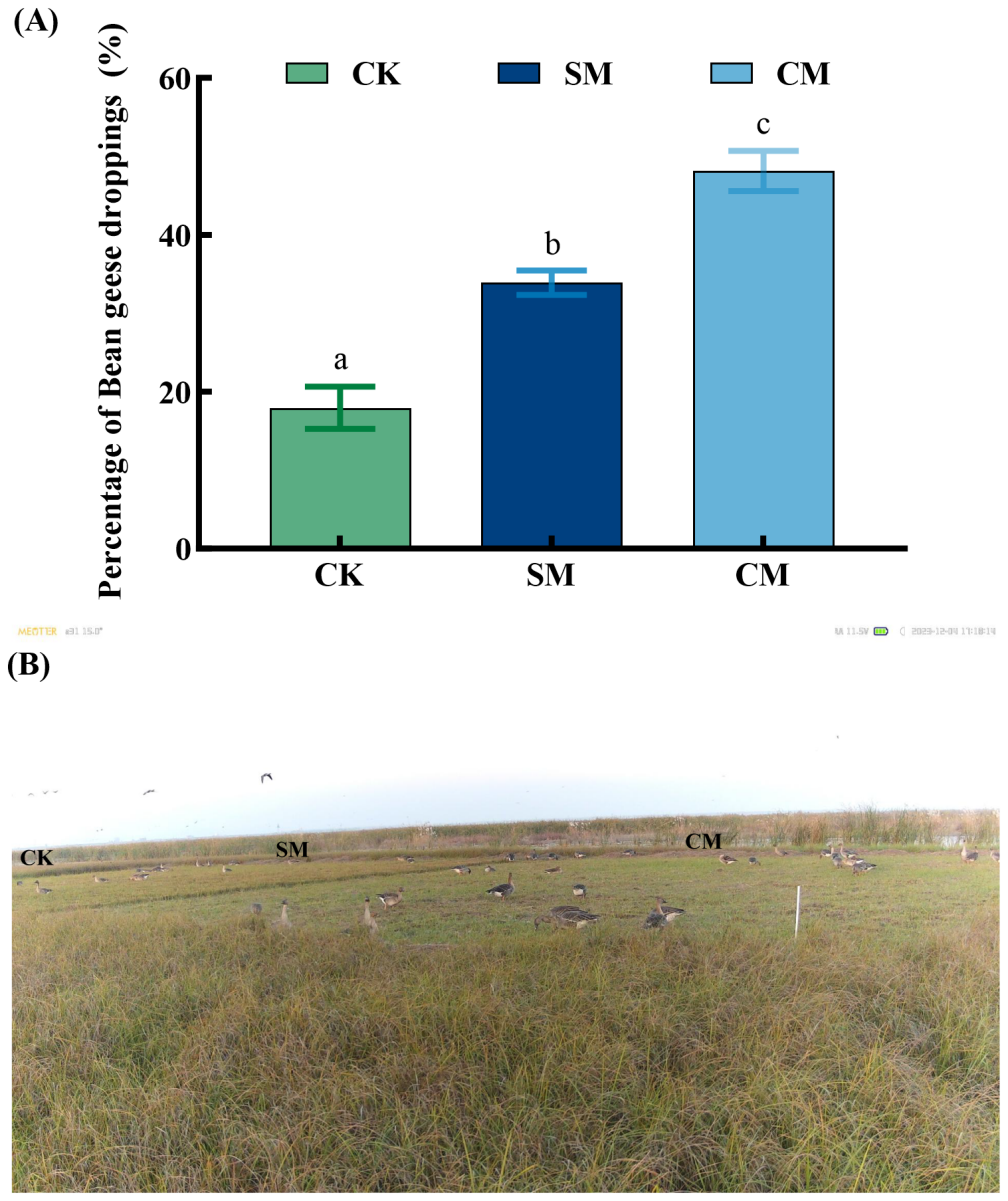
**(A)** Percentage of goose droppings in quadrats with different mowing treatments, November 2023-March 2024. **(B)** A photograph captured by an infrared camera showing Bean Geese foraging in the sampling site. The data are expressed as the mean ± standard error (SE). Different letters indicate significant differences at the 0.05 significance level. Different lowercase letters denote significant differences among treatments with a *p* < 0.05.

Bean geese, the dominant overwintering goose species in Dongting Lake, exhibit strong trophic reliance on *C. brevicuspis* (Zuo et al., 2018). Based on previous research (Zhang et al., 2020), we analyzed the utilization of *C. brevicuspis* under different mowing treatments. The results showed significant differences in the percentage of food utilization by Bean geese among CM, SM, and CK quadrats during the overwintering period (*p* < 0.001, **Figure 8A**). The number of Bean geese droppings was highest in CM quadrats, followed by SM quadrats, and lowest in CK treatment. This indicates that mowing, particularly CM, significantly increased the utilization rate of *C. brevicuspis* by Bean geese (**Figures 8A, B**), which is likely closely related to the significant improvement in *C. brevicuspis* quality following mowing (**Figures 3A–C**).

## 4 Discussion

### 4.1 Mechanisms of mowing-induced improvement in food quality for herbivorous geese

Our study showed that mowing improves the food quality for herbivorous geese through the synergistic effects of compensatory growth and clonal growth strategies. Mowing significantly improved the food quality for herbivorous geese, manifested by increased moisture and nitrogen content of *C. brevicuspis* coupled with decreased crude fiber content (**Figures 3A–C**). These observations align with findings from previous studies (Mikhailova et al., 2000; Gardarin et al., 2014). Increased leaf nitrogen content generally reflects higher chlorophyll concentrations and enhanced photosynthetic rates (Ziter and MacDougall, 2013), the elevated leaf nitrogen content in *C. brevicuspis* likely results from resource reallocation induced by compensatory growth, which stimulates the synthesis of proteins and nucleic acids (Hou et al., 2020). These biomolecules are crucial for restoring photosynthetic machinery, thereby enhancing both leaf nutritional quality for herbivorous geese. Concomitantly, the mowing-induced decline in crude fiber directly improved food digestibility for herbivorous geese, as crude fiber content relates negatively to digestibility of food (Waghorn and Clark, 2004). This process is intrinsically linked to the plant’s clonal growth strategy, *C. brevicuspis* accelerates the repair of photosynthetic organs and the synthesis of metabolites through rapid activation of its belowground bud banks, particularly short rhizome buds (SRBs). The synergistic improvement of both nutrient availability and food digestibility provides a mechanistic explanation for the elevated defecation rate observed in Bean geese utilizing mowed vegetation quadrats (**Figures 5A, B**).

Mowing significantly reduced height, density, and aboveground biomass of *C. brevicuspis* compared to CK treatment (**Figures 4A–C**), our study also showed that the height of *C. brevicuspis* in mowing treatments was consistently lower than CK treatment throughout the experiment (**Figure 4A**), attributable to both direct removal of photosynthetic tissues (Liu et al., 2023) and positive feedback from geese preferentially foraging on shorter vegetation (Cong et al., 2012; Fox et al., 2013), which is consistent with the results of this study (**Figures 8A, B**). Herbivorous geese preferentially foraged in the mowed quadrats, subjecting *C. brevicuspis* to repeated mowing pressure, which slowed its growth during recovery and regeneration, further influencing its overall growth.

The ability of plants to regenerate and grow after mowing is likely closely related to carbon reserves in perennial organs (Deng et al., 2013a). Bud formation requires energy and carbon allocation, and in response to mowing stress, *C. brevicuspis* may prioritize resource allocation to increase nutritional content (e.g., nitrogen and moisture content, **Figures 3A, B**). Following mowing, plants may allocate more resources to bud survival and regeneration, rather than to the growth and development of new buds, leading to reduction in bud biomass (**Figures 7A, B**). Moreover, although root biomass showed no significant changes (**Table 2**), root growth is still affected by resource allocation. Mowing reduces soil organic matter by decreasing aboveground biomass (Snyder and Williams, 2003), thereby limiting the return of plant residues to the soil and decreasing the transfer of photosynthetic carbon to roots (Wei et al., 2016). Reduced carbon accumulation and energy supply may cause plants to allocate more resources to aboveground regeneration, restoring photosynthesis under mowing stress (Mikola et al., 2009). This adjustment may explain the relative stability and delayed response of root systems to external disturbances, as root biomass in the complete mowing treatment was slightly lower than in other treatments (**Figure 4D**).

Under persistent mowing pressure, *C. brevicuspis* maintained both density homeostasis and compositional homeostasis in its belowground bud bank. However, this study involved only a single winter season (2023/2024) of mowing experiments; whether frequent mowing would induce degradation of *Carex* species remains to be further investigated. Belowground bud bank regeneration depends on the number and location of dormant and viable buds stored on belowground organs (Bombo et al., 2022). The recovery of bud banks of *C. brevicuspis* after mowing is driven by two key mechanisms: (1) rapid regeneration of short rhizome buds (SRB), which prioritize vertical tillering, and (2) horizontal expansion of long rhizome buds (LRB) (Ott and Hartnett, 2015; Chen and Deng, 2016). This structural allow clonal plants to regenerate and persist after disturbances, thereby promoting population resilience (Chen and Deng, 2016). For *C. brevicuspis*, mowing did not significantly affect the density of its belowground bud banks (**Figures 5A–C**), which may be closely related to its unique survival and regeneration mechanisms. *C. brevicuspis* not only survives mowing but also activates dormant buds while regenerating photosynthetic tissues (Ott and Hartnett, 2011), this coordinated response enhances resource allocation efficiency, thereby accelerating post-disturbance recovery (Dalgleish and Hartnett, 2009). Despite reduced biomass per ramet (**Figures 7A, B**), the stable bud density (**Figures 5A–C**) suggests that *C. brevicuspis* compensates for biomass loss through accelerated activation of dormant buds rather than bud neogenesis. Moreover, this adaptation maintains bud bank composition stability under mowing treatments (**Figure 6**), as both SRB and LRB experience comparable growth constraints while effectively contributing to vegetative regeneration. Therefore, although subjected to disturbance pressure, *C. brevicuspis* sustains population persistence through its bud bank recovery.

### 4.2 Ecological trade-offs and management implications of long-term mowing

Hydrological regimes significantly affect waterbirds’ foraging behavior, diversity, and abundance by altering habitat availability and vegetation conditions (Xia et al., 2017; Liu et al., 2024b). Study has shown that the spatial distribution of Lesser White-fronted Geese in East Dongting Lake was significantly correlated with hydrological regimes (Zhang et al., 2018), where annual changes in hydrological conditions may exacerbate waterbird population distributions differences across years. Early water recession often triggers habitat shifts, driving geese to forage on mudflats and exploit alternative food resources such as dicotyledons and mosses (Zhang et al., 2020). Water level fluctuations directly affect the composition, distribution, and productivity of wetland vegetation (Guan et al., 2014; Gao et al., 2020; Wang et al., 2023), which, in turn, influence the populations of herbivorous geese dependent on these plants for food (Zhang et al., 2021). Seasonal water recession timing and magnitude in East Dongting Lake critically determine the exposure duration of meadows and successional trajectories of plant communities (Cong et al., 2012). If the water level drops too early in autumn (**Figure 2**), plants like *C. brevicuspis* may age prematurely, reducing their quality by the time geese arrive in early November. This decline forces geese to feed on older-growth plants (Cong et al., 2012). Herbivorous geese then face sedge meadows with low crude protein and high fiber content (Zhang et al., 2021), exacerbating mismatches between food quality and their energy demands (Guan et al., 2014; Sun et al., 2020). Conversely, delayed water recession suppresses plant germination, resulting in winter food scarcity (Zhang et al., 2020). Consequently, adaptive management strategies integrating hydrological variability must be prioritized in wetland conservation to ensure both food supply and habitat quality for herbivorous geese.

While mowing improves short-term food quality through compensatory growth, frequent interventions risk depleting carbon reserves and impairing bud bank functionality. After mowing disturbances, plants prioritize carbon and nutrient allocation to increase nitrogen and moisture content, accelerating the recovery of photosynthesis and growth to ensure their growth potential. Continuous mowing may gradually deplete carbon reserves and bud banks, thereby reducing recovery capacity (N’Guessan et al., 2011). Our results showed that mowing significantly reduces the biomass of rhizome buds per ramet (**Figures 7A, B**), which may impact the density of belowground bud banks over the long term, consequently affect *C. brevicuspis* population density. Moreover, stubble height determines the retention of photosynthetic organs (leaves), enabling continued post-mowing photosynthesis to fuel regeneration. This process supplies energy and carbon to belowground structures (e.g., bud banks and roots), alleviating their stress (Wang et al., 2015). Stubble height is also a critical factor for the response of plant nutrient concentration to mowing (Yang et al., 2020). The retention of stubble height is pivotal for balancing short-term food quality and long-term ecosystem stability. Moderate stubble heights preserve partial photosynthetic tissues, sustaining post-mowing carbon fixation to fuel root and bud bank recovery (Wang et al., 2015). Appropriate mowing intensity can enhance nutrients concentration of plants and population stability (Ji et al., 2021). Studies in Inner Mongolian steppes confirmed that heavy mowing (stubble <6 cm) suppresses plant biomass recovery by damaging photosynthetic organs and growth points, while light mowing (stubble >12 cm) optimizes both productivity and nutrient retention (Yang et al., 2020). This residual biomass mitigates soil carbon loss by maintaining organic matter input, thereby stabilizing microbial activity and nutrient cycling (Snyder and Williams, 2003; Ziter and MacDougall, 2013). A 4-year mowing experiment in *Leymus chinensis* grasslands also demonstrated that stubble heights of 6–12 cm enhanced plant nutrient concentrations without significantly altering soil organic carbon or total nitrogen pools, suggesting a decoupling between rapid plant responses and slower soil nutrient dynamics (Yang et al., 2020). Conversely, ground-level mowing (stubble <0.3 cm) reduced soil organic carbon by 7.3% over four years due to diminished root exudation and litter decomposition, highlighting the risks of carbon reserve depletion under intensive management (Liu et al., 2023).

Therefore, to alleviate the mismatch between food availability and the arrival timing of herbivorous geese caused by hydrological changes, it is essential to scientifically regulate mowing frequency and intensity. This approach aims to enhance food quality for herbivorous geese while ensuring their sustained growth and recovery capacity. These combined effects maintain the stability of wetland ecosystems and the sustainability of waterbird habitats.

## 5 Conclusion and recommendations

This study explored the role of mowing in improving the food quality for herbivorous geese in Dongting Lake and its underlying mechanisms through a mowing control experiment. The results showed that mowing significantly improved the food quality of *C. brevicuspis*, optimizing its palatability and nutritional value, thus providing more abundant food resources for herbivorous geese. Mowing improves the food quality for herbivorous geese through the synergistic effects of compensatory growth and clonal growth strategies. Mowing increased the nitrogen and moisture content of *C. brevicuspis* while reducing the crude fiber content. Simultaneously, mowing significantly reduced the density, aboveground biomass, and belowground bud biomass of *C. brevicuspis*, which may affect its long-term recovery potential. Therefore, this study demonstrates that mowing is an effective management measure to enhance the food availability for herbivorous geese during the overwintering period. However, wetland management and conservation should carefully consider mowing frequency and intensity and adjust them according to hydrological changes and the needs of herbivorous geese. Such a management strategy not only improves food quality but also ensures the long-term growth and recovery of plants, thereby supporting the sustainability of wetland vegetation and the food supply for herbivorous geese.

## Data Availability

The datasets presented in this study can be found in online repositories. The names of the repository/repositories and accession number(s) can be found in the article/**Supplementary Material**.

## References

[B1] Beard K. H. Choi R. T. Leffler A. J. Carlson L. G. Kelsey K. C. Schmutz J. A.. (2019). Migratory goose arrival time plays a larger role in influencing forage quality than advancing springs in an Arctic coastal wetland. PLoS One 14, e0213037. doi: 10.1371/journal.pone.0213037 30865725 PMC6415786

[B2] Benson E. J. Hartnett D. C. Mann K. H. (2004). Belowground bud banks and meristem limitation in tallgrass prairieplant populations. Am. J. Bot. 91, 416–421. doi: 10.3732/ajb.91.3.416 21653397

[B3] Cao L. Barter M. Lei G. (2008). New Anatidae population estimates for eastern China: Implications for current flyway estimates. Biol. Conserv. 141, 2301–2309. doi: 10.1016/j.biocon.2008.06.022

[B4] Chen X. Deng Z. (2016). Consequences of repeated defoliation on belowground bud banks of *Carex brevicuspis* (Cyperaceae) in the dongting lake wetlands, China. Front. Plant Sci. 7. doi: 10.3389/fpls.2016.01119 PMC4965449 27524993

[B5] Chen X. Deng Z. Xie Y. Li F. Hou Z. Li X. (2014). Demography of *Carex brevicuspis* (Cyperaceae) rhizome populations: a wetland sedge that produces both elongated and shortened rhizomes. Nordic J. Bot. 32, 251–256. doi: 10.1111/j.1756-1051.2013.00094.x

[B6] Chen X. Xie Y. Deng Z. Li F. Hou Z. (2011). A change from phalanx to guerrilla growth form is an effective strategy to acclimate to sedimentation in a wetland sedge species *Carex brevicuspis* (Cyperaceae). Flora - Morphology Distribution Funct. Ecol. Plants 206, 347–350. doi: 10.1016/j.flora.2010.07.006

[B7] Cheng J. Xu L. Wang X. Jiang J. You H. (2018). Assessment of hydrologic alteration induced by the Three Gorges Dam in Dongting Lake, China. River Res. Apps 34, 686–696. doi: 10.1002/rra.3297

[B8] Cong P. Wang X. Cao L. Fox A. D. (2012). Within-Winter Shifts in Lesser White-Fronted Goose *Anser erythropus* Distribution at East Dongting Lake, China. Ardea 100, 5–11. doi: 10.5253/078.100.0103

[B9] Dalgleish H. J. Hartnett D. C. (2009). The effects of fire frequency and grazing on tallgrass prairie productivity and plant composition are mediated through bud bank demography. Plant Ecol. 201, 411–420. doi: 10.1007/s11258-008-9562-3

[B10] Deng Z. Chen X. Xie Y. Li X. Pan Y. Li F. (2013a). Effects of size and vertical distribution of buds on sprouting and plant growth of the clonal emergent macrophyte *Miscanthus sacchariflorus* (Poaceae). Aquat. Bot. 104, 121–126. doi: 10.1016/j.aquabot.2012.08.004

[B11] Deng Z. Chen X. Xie Y. Pan Y. Li F. Hou Z.. (2013b). Plasticity of the clonal growth in the wetland sedge *carex brevicuspis* along a small-scale elevation gradient in dongting lake wetlands, China. Annales Botanici Fennici 50, 151–159. doi: 10.5735/085.050.0305

[B12] Deng Z. Chen X. Xie Y. Xie Y. Hou Z. Li F. (2015). The role of seedling recruitment from juvenile populations of *Carex brevicuspis* (Cyperaceae) at the Dongting Lake wetlands, China. Sci. Rep. 5, 8646. doi: 10.1038/srep08646 25728624 PMC5155402

[B13] Durant J. Hjermann D. Ottersen G. Stenseth N. (2007). Climate and the match or mismatch between predator requirements and resource availability. Clim. Res. 33, 271–283. doi: 10.3354/cr033271

[B14] Feng D. Guan L. Shi L. Zeng Q. Liu X. Zhang H.. (2014). Impact of autumn hydrologic regime on plants in beach and distribution of populations of wintering lesser white-fronted goose in East Dongting Lake. Wetland Sci. 12, 491–498. doi: 10.13248/j.cnki.wetlandsci.2014.04.013

[B15] Feng L. Hu C. Chen X. Zhao X. (2013). Dramatic inundation changes of China’s two largest freshwater lakes linked to the Three Gorges Dam. Environ. Sci. Technol. 47, 9628–9634. doi: 10.1021/es4009618 23919680

[B16] Fox A. D. Lei C. Barter M. Rees E. C. Hearn R. D. Hao C. P. (2013). The functional use of East Dongting Lake, China, by wintering geese. Wildfowl 58, 3–19.

[B17] Gao Y. Li Y. Zou D. (2020). Hydrological regime change and its ecological responses in East Dongting Lake, China. Ecohydrology Hydrobiology 20, 142–150. doi: 10.1016/j.ecohyd.2019.07.003

[B18] Gardarin A. Garnier E. Carrère P. Cruz P. Andueza D. Bonis A.. (2014). Plant trait-digestibility relationships across management and climate gradients in permanent grasslands. J. Appl. Ecol. 51, 1207–1217. doi: 10.1111/1365-2664.12293

[B19] Guan L. Wen L. Feng D. Zhang H. Lei G. (2014). Delayed flood recession in central yangtze floodplains can cause significant food shortages for wintering geese: results of inundation experiment. Environ. Manage. 54, 1331–1341. doi: 10.1007/s00267-014-0350-7 25164981

[B20] Hou D. Guo K. Liu C. (2020). Asymmetric effects of grazing intensity on macroelements and microelements in grassland soil and plants in Inner Mongolia Grazing alters nutrient dynamics of grasslands. Ecol. Evol. 10, 8916–8926. doi: 10.1002/ece3.6591 32884667 PMC7452780

[B21] Huhta A.-P. Lennartsson T. Tuomi J. Rautio P. Laine K. (2000). Tolerance of Gentianella campestris in relation to damage intensity: an interplay between apical dominance and herbivory. Evolutionary Ecol. 14, 373–392. doi: 10.1023/A:1011028722860

[B22] Ji G. Li B. Yin H. Liu G. Yuan Y. Cui G. (2021). Non-utilization is not the best way to manage lowland meadows in Hulun Buir. Front. Plant Sci. 12. doi: 10.3389/fpls.2021.704511 PMC8322850 34335668

[B23] Kołos A. Banaszuk P. (2013). Mowing as a tool for wet meadows restoration: Effect of long-term management on species richness and composition of sedge-dominated wetland. Ecol. Eng. 55, 23–28. doi: 10.1016/j.ecoleng.2013.02.008

[B24] Lai X. Jiang J. Huang Q. (2013). Effects of the normal operation of the Three Gorges Reservoir on wetland inundation in Dongting Lake, China: a modelling study. Hydrological Sci. J. 58, 1467–1477. doi: 10.1080/02626667.2013.831418

[B25] Liu J. Li L. Ji L. Li Y. Liu J. Li Y. (2023). Divergent effects of grazing versus mowing on plant nutrients in typical steppe grasslands of Inner Mongolia. J. Plant Ecol. 16, rtac032. doi: 10.1093/jpe/rtac032

[B26] Liu Y. Li J. Yan D. Chen L. Li M. Luan Z. (2024a). Typical vegetation dynamics and hydrological changes of Dongting Lake wetland from 1985 to 2020. Ecohydrology Hydrobiology 24, 910–919. doi: 10.1016/j.ecohyd.2023.04.008

[B27] Liu Y. Marnn P. Jiang H. Wen Y. Yan H. Li D.. (2024b). A study on the response of waterbird diversity to habitat changes caused by ecological engineering construction. Ecol. Eng. 208, 107358. doi: 10.1016/j.ecoleng.2024.107358

[B28] Mikhailova E. A. Bryant R. B. Cherney D. J. R. Post C. J. Vassenev I. I. (2000). Botanical composition, soil and forage quality under different management regimes in Russian grasslands. Agriculture Ecosystems Environ. 80, 213–226. doi: 10.1016/S0167-8809(00)00148-1

[B29] Mikola J. Setälä H. Virkajärvi P. Saarijärvi K. Ilmarinen K. Voigt W.. (2009). Defoliation and patchy nutrient return drive grazing effects on plant and soil properties in a dairy cow pasture. Ecol. Monogr. 79, 221–244. doi: 10.1890/08-1846.1

[B30] Molina C. D. Tognetti P. M. Graff P. Chaneton E. J. (2021). Mowing does not redress the negative effect of nutrient addition on alpha and beta diversity in a temperate grassland. J. Ecol. 109, 1501–1510. doi: 10.1111/1365-2745.13573

[B31] Murray N. J. Fuller R. A. (2015). Protecting stopover habitat for migratory shorebirds in East Asia. J. Ornithol 156, 217–225. doi: 10.1007/s10336-015-1225-2

[B32] N’Guessan M. Hartnett D. C. Hartnett D. C. Hartnett D. C. (2011). Differential responses to defoliation frequency in Little Bluestem (*Schizachyrium scoparium*) in tallgrass prairie: Implications for herbivory tolerance and avoidance. Plant Ecol. 212, 1275–1285. doi: 10.1007/s11258-011-9904-4

[B33] O’neal B. J. Heske E. J. Stafford J. D. (2008). Waterbird response to wetlands restored through the conservation reserve enhancement program. J. Wildlife Manage. 72, 654–664. doi: 10.2193/2007-165

[B34] Ott J. P. Hartnett D. C. (2011). Bud production and dynamics of flowering and vegetative tillers in *Andropogon gerardii* (Poaceae): the role of developmental constraints. Am. J. Bot. 98, 1293–1298. doi: 10.3732/ajb.1000264 21788531

[B35] Ott J. P. Hartnett D. C. (2015). Bud bank dynamics and clonal growth strategy in the rhizomatous grass, Pascopyrum smithii. Plant Ecol. 216, 395–405. doi: 10.1007/s11258-014-0444-6

[B36] Snyder K. A. Williams D. G. (2003). Defoliation alters water uptake by deep and shallow roots of *Prosopis velutina* (Velvet mesquite). Funct. Ecol. 17, 363–374. doi: 10.1046/j.1365-2435.2003.00739.x

[B37] Sosnová M. van Diggelen R. Klimešová J. (2010). Distribution of clonal growth forms in wetlands. Aquat. Bot. 92, 33–39. doi: 10.1016/j.aquabot.2009.09.005

[B38] Sun G. Lei G. Qu Y. Zhang C. He K. (2020). The operation of the three gorges dam alters wetlands in the middle and lower reaches of the yangtze river. Front. Environ. Sci. 8. doi: 10.3389/fenvs.2020.576307

[B39] Teng J. Xia S. Duan H. Liu Y. Yu X. Yang W. (2023). Temporal and spatial evolution of herbivorous waterbird habitat in floodplain wetland driven by hydrology. Water Resour. Res. 59, e2022WR034399. doi: 10.1029/2022WR034399

[B40] Van De Koppel J. Huisman J. van der Wal R. Olff H. (1996). Patterns of herbivory along a prouductivity gradient: an empirical and theoretical investigation. Ecology 77, 736–745. doi: 10.2307/2265498

[B41] Van Der Graaf A. J. Stahl J. Veen G. F. Havinga R. M. Drent R. H. (2007). Patch choice of avian herbivores along a migration trajectory–From Temperate to Arctic. Basic Appl. Ecol. 8, 354–363. doi: 10.1016/j.baae.2006.07.001

[B42] Veloso C. Bozinovic F. (1993). Dietary and digestive constraints on basal energy metabolism in a small herbivorous rodent. Ecology 74, 2003–2010. doi: 10.2307/1940843

[B43] Waghorn G. C. Clark D. A. (2004). Feeding value of pastures for ruminants. N. Z. Vet. J. 52, 320–331. doi: 10.1080/00480169.2004.36448 15768132

[B44] Wang X. Fox A. D. Cong P. Barter M. Cao L. (2012). Changes in the distribution and abundance of wintering Lesser White-fronted Geese *Anser erythropus* in eastern China. Bird Conserv. Int. 22, 128–134. doi: 10.1017/S095927091100030X

[B45] Wang M. Gu Q. Liu G. Shen J. Tang X. (2019). Hydrological condition constrains vegetation dynamics for wintering waterfowl in China’s east dongting lake wetland. Sustainability 11, 4936. doi: 10.3390/su11184936

[B46] Wang L. Wei L. Wang J. Gan Y. Wu Y. (2015). The compensatory growth of plant community, synusia and species under different clipping intensity. Acta Prataculturae Sin. 24, 35. doi: 10.11686/cyxb2014473

[B47] Wang C. Xia S. Yu X. Li W. (2024). It takes two to Tango: Plant height and nutrient level determine the diet selection of wintering geese in Poyang Lake, a Ramsar wetland. Global Ecol. Conserv. 49, e02802. doi: 10.1016/j.gecco.2024.e02802

[B48] Wang H. Zhang X. Xu Y. Wang H. Song M. Shen Y. (2023). Ecological regulation of water level should be combined with seed supplementation for lakeshore *Carex* community restoration in Yangtze-disconnected lakes. Sci. Total Environ. 897, 165358. doi: 10.1016/j.scitotenv.2023.165358 37419353

[B49] Wang X. Zhang Y. Zhao M. Cao L. Fox A. D. (2013). The benefits of being big: effects of body size on energy budgets of three wintering goose species grazing *Carex* beds in the Yangtze River floodplain, China. J. Ornithol 154, 1095–1103. doi: 10.1007/s10336-013-0979-7

[B50] Washburn B. E. Seamans T. W. (2012). Foraging preferences of Canada geese among turfgrasses: Implications for reducing human–goose conflicts. J. Wildl Manag 76, 600–607. doi: 10.1002/jwmg.293

[B51] Wei J. Liu W. Wan H. Cheng J. Li W. (2016). Differential allocation of carbon in fenced and clipped grasslands: a ^13^C tracer study in the semiarid Chinese Loess Plateau. Plant Soil 406, 251–263. doi: 10.1007/s11104-016-2879-0

[B52] Wu H. Zeng G. Liang J. Chen J. Xu J. Dai J.. (2017). Responses of landscape pattern of China’s two largest freshwater lakes to early dry season after the impoundment of Three-Gorges Dam. Int. J. Appl. Earth Observation Geoinformation 56, 36–43. doi: 10.1016/j.jag.2016.11.006

[B53] Xia S. Liu Y. Chen B. Jia Y. Zhang H. Liu G.. (2017). Effect of water level fluctuations on wintering goose abundance in Poyang Lake wetlands of China. Chin. Geogr. Sci. 27, 248–258. doi: 10.1007/s11769-016-0840-z

[B54] Xie Y. Tang Y. Chen X. Li F. Deng Z. (2015). The impact of Three Gorges Dam on the downstream eco-hydrological environment and vegetation distribution of East Dongting Lake. Ecohydrology 8, 738–746. doi: 10.1002/eco.1543

[B55] Yang Z. Minggagud H. Baoyin T. Li F. Y. (2020). Plant production decreases whereas nutrients concentration increases in response to the decrease of mowing stubble height. J. Environ. Manage. 253, 109745. doi: 10.1016/j.jenvman.2019.109745 31671323

[B56] Zhang P. Zou Y. Xie Y. Zhang S. Chen X. Li F.. (2020). Hydrology-driven responses of herbivorous geese in relation to changes in food quantity and quality. Ecol. Evol. 10, 5281–5292. doi: 10.1002/ece3.6272 32607151 PMC7319142

[B57] Zhang P. Zou Y. Xie Y. Zhang H. Liu X. Gao D.. (2018). Shifts in distribution of herbivorous geese relative to hydrological variation in East Dongting Lake wetland, China. Sci. Total Environ. 636, 30–38. doi: 10.1016/j.scitotenv.2018.04.247 29702400

[B58] Zhang P. Zou Y. Xie Y. Zhang S. Zhu F. Chen X.. (2021). Phenological mismatch caused by water regime change may explain the population variation of the vulnerable lesser white-fronted goose in east Dongting Lake, China. Ecol. Indic. 127, 107776. doi: 10.1016/j.ecolind.2021.107776

[B59] Zhang P. Zhang S. Zou Y. Wu T. Li F. Deng Z. (2023). Integrating suitable habitat dynamics under typical hydrological regimes as guides for the conservation and restoration of different waterbird groups. J. Environ. Man. 345, 118451. doi: 10.1016/j.jenvman.2023.118451 37385199

[B60] Zhao W. Chen S. Lin G. (2008). Compensatory growth responses to clipping defoliation in *Leymus chinensis* (Poaceae) under nutrient addition and water deficiency conditions. Plant Ecol. 196, 85–99. doi: 10.1007/s11258-007-9336-3

[B61] Ziter C. MacDougall A. S. (2013). Nutrients and defoliation increase soil carbon inputs in grassland. Ecology 94, 106–116. doi: 10.1890/11-2070.1 23600245

[B62] Zou Y. Tang Y. Li Y. Zhao Q. Zhang H. (2017). Response of herbivorous geese to wintering habitat changes: conservation insights from long-term population monitoring in the East Dongting Lake, China. Reg. Environ. Change 17, 879–888. doi: 10.1007/s10113-016-1087-z

[B63] Zuo O. Lei J. Wang Y. Ma T. Lu C. Wen L.. (2018). Comparison of the Growth Indexes of *Carex brevicuspis* in Habitat under Different Management Ways for Wintering Geese in East Dongting Lake. Wetland Sci. 16, 537–545. doi: 10.13248/j.cnki.wetlandsci.2018.04.013

